# Kaempferol mitigates reproductive dysfunctions induced by *Naja nigricollis* venom through antioxidant system and anti-inflammatory response in male rats

**DOI:** 10.1038/s41598-024-54523-w

**Published:** 2024-02-16

**Authors:** Akindele Oluwatosin Adeyi, Babafemi Siji Ajisebiola, Akanni Abubakar Sanni, Johnson Olaleye Oladele, Abdur-Rahman Kolawole Mustapha, Omotayo Opemipo Oyedara, Olukunle Silas Fagbenro

**Affiliations:** 1 Animal Physiology Unit, Department of Zoology, University of Ibadan, Ibadan, Nigeria; 2 Department of Zoology, Osun State University, Osogbo, Nigeria; 3 Biochemistry Unit, Department of Chemical Sciences, Kings University, Ode-Omu, Nigeria; 4 Department of Biotechnology, Osun State University, Osogbo, Nigeria; 5 Cell Biology and Genetics Unit, Department of Zoology, University of Ibadan, Ibadan, Nigeria

**Keywords:** Toxicology, Proteins

## Abstract

*Naja nigricollis* Venom (NnV) contains complex toxins that affects various vital systems functions after envenoming. The venom toxins have been reported to induce male reproductive disorders in envenomed rats. This present study explored the ameliorative potential of kaempferol on NnV-induced male reproductive toxicity. Fifty male wistar rats were sorted randomly into five groups (n = 10) for this study. Group 1 were noted as the control, while rats in groups 2 to 5 were injected with LD_50_ of NnV (1.0 mg/kg bw; i.p.). Group 2 was left untreated post envenomation while group 3 was treated with 0.2 ml of polyvalent antivenom. Groups 4 and 5 were treated with 4 and 8 mg/kg of kaempferol, respectively. NnV caused substantial reduction in concentrations of follicle stimulating hormone, testosterone and luteinizing hormone, while sperm motility, volume and counts significantly (p < 0.05) decreased in envenomed untreated rats. The venom enhanced malondialdehyde levels and substantially decreased glutathione levels, superoxide dismutase and glutathione peroxidase activities in the testes and epididymis of envenomed untreated rats. Additionally, epididymal and testicular myeloperoxidase activity and nitric oxide levels were elevated which substantiated severe morphological defects noticed in the reproductive organs. However, treatment of envenomed rats with kaempferol normalized the reproductive hormones with significant improvement on sperm functional parameters. Elevated inflammatory and oxidative stress biomarkers in testis and epididymis were suppressed post kaempferol treatment. Severe histopathological lesions in the epididymal and testicular tissues were ameliorated in the envenomed treated groups. Results highlights the significance of kaempferol in mitigating reproductive toxicity induced after snakebite envenoming.

## Introduction

Snakebite envenoming is acknowledged as a neglected tropical health challenge that poses a serious public risk and is projected to cause 81,000–138,000 deaths annually from 1.8 million cases out of 2.7 million globally^1,2^. Due of the nature of their occupations, rural residents in underdeveloped areas of Asia and sub-Saharan Africa were often the victims of snakebite envenomation^3,4^. Since most people in these locations are farmers, nomads, or hunters, there is a higher likelihood of coming into contact with snakes^5^. An estimated 1,000,000 snake bites occur annually in Africa, mostly in the sub-Saharan region. These bites result in 100,000 to 500,000 envenomations and 10,000 to 30,000 fatalities annually^5,6^.

Snakebite envenomation caused by *Bitis arietans* (Puff adder), *Naja nigricollis* (black-necked spitting cobra), and *Echis ocellatus* (West African carpet viper) is a major medical burden in the sub-Saharan region^7–9^. Among these African snakes, *N. nigricollis*, belonging to the Elapidae family, is particularly dangerous. It may project a venom spray from its fangs, and its bites can frequently result in severe systemic damage and even death^10–12^. These attributes made the World Health Organization considered *N. nigricollis* as Category 1 of venomous snake species^13^.

*N. nigricollis* Venom (NnV) is composed of toxic proteins mainly phospholipases A2, three-finger toxins, cytotoxins and neurotoxins among others^14^. These toxic proteins are largely responsible for severe cytotoxic and neurotoxic manifestations after *N. nigricollis* envenomation^7,14^. The combined action of many toxins acting additively or synergistically on different important organs in envenomed victims is thought to be responsible for the overall pathophysiological effect of *N. nigricollis* envenomation^15^. Snake venom toxins have been shown to have a significant impact on several vital organ systems, with the reproductive, endocrine, cardiovascular, and neurological systems being their primary targets^11,16,17^. Research has shown that NnV causes endocrine and reproductive toxicities in vivo, which severely impair the physiological functions of important reproductive organs^10,11^.

An intravenous injection of a specific snake antivenom is the recommended course of treatment for envenomation caused by *N. nigricollis*. However, there are a number of drawbacks to antivenom treatment, including its scarcity, high cost, difficulty in preservation due to remote regions' lack of electricity, and combination of clinical side effects after use^18^. As a result, the majority of rural residents rely on sometimes ineffective alternative treatments such as medicinal herbs. To prevent organ systems fatalities following envenoming, effective treatment of snakebite envenoming necessitates a thorough approach and prompt availability to appropriate treatment. Thus, antivenom researchers are very interested in developing an effective, economical alternative with minimal limitations.

Studies have validated the potency of numerous medicinal plants as antivenom agents against several snake species^10,19,20^. However, the primary antivenom bioactive compounds of these plants have only been partially described by researchers. Kaempferol was discovered as the primary antivenom compound in *Moringa oleifera* against snake venom toxicity in our most recent study^21^. Kaempferol is a natural flavonoid that has been shown to have the capacity to initiates a variety of bioactivities against several prevalent health related issues^22,23^. Our previous investigation observed that NnV induced reproductive abnormalities through oxidative damage and pro-inflammatory cytokine modulation in male rats^11^. On the other hand, there is no information currently available about the amelioration of NnV-induced male reproductive pathophysiologies. Thus, the purpose of this study was to provide valuable information about the ameliorative effect of kaempferol against male reproductive damage caused by NnV in rats.

## Results

### Clinical signs of toxicity, body weight gain, organ weight changes, and organo-somatic index of experimental rats

Following envenomation, symptoms such as reduced hunger, restricted movement, dizziness, and fatality are indicative of toxicity. The envenomed untreated group recorded five deaths after envenomation on day 1, 5, 20, and 41, however, the control group did not record any mortality (Table 1). Three deaths were observed in each of the envenomed groups treated with 0.2 ml of antivenom and 4 mg/kg of kaempferol whereas, no mortality was noted in the envenomed group treated with 8 mg/kg of kaempferol. The control rats' body weight gain was considerably (p < 0.05) greater than that of the envenomed untreated and treated rats. In contrast to the envenomed treated groups, the body weight gain of the envenomed untreated group is significantly (p < 0.05) lower (Table 2). Testicular weights of envenomed untreated rats were significantly (p < 0.05) lower than those of the control and envenomed treated groups, while the experimental rats' testiculo-somatic index showed a similar trend (Table 2).

**Table 1:** Mortality observed in envenomed group during experimental period.

Groups	Envenomation	Number of death					Mortality (%)
		Day 1	Day 5	Day 20	Day 41	Day 50	
1	–	–	–	–	–	–	0.00
2	–	1	2	1	1	–	50.00
3	–	–	1	1	1	–	30.00
4	–	–	1	–	1	–	20.00
5	–	–	–	–	–	–	0.00

Number of rats per group (n = 10).

Group 1: Injected with saline (Control), Group 2: Envenomed not treated (Venom control), Group 3: Envenomed and treated with 0.2 ml of antivenom, Group 4: Envenomed and treated with 4 mg/kg^−1^ of kaempferol, Group 5: Envenomed and treated with 8 mg/kg^−1^ of kaempferol.

**Table 2:** Body weight gain and organo-somatic index of experimental rats.

Groups	Body weight gain (g)	Testicular weight (g)	Testiculosomatic index (%)
1	10.17 ± 0.31^a^	1.83 ± 0.04^a^	2.96 ± 0.62^a^
2	4.54 ± 0.23^c^	1.32 ± 0.02^c^	2.04 ± 0.17^c^
3	8.62 ± 0.06^b^	1.77 ± 0.11^b^	2.60 ± 0.10^b^
4	8.82 ± 0.11^b^	1.73 ± 0.12^b^	2.71 ± 0.22^b^
5	9.12 ± 0.10^b^	1.80 ± 0.12^a^	2.82 ± 0.02^a^

Data are represented as mean ± SE (n = 5). Mean with different lower-case letter represent significant difference among the groups at p < 0.05 using DMRT.

Group 1: Injected with saline (Control), Group 2: Envenomed not treated (Venom control), Group 3: Envenomed and treated with 0.2 ml of antivenom, Group 4: Envenomed and treated with 4 mg/kg^−1^ of kaempferol, Group 5: Envenomed and treated with 8 mg/kg^−1^ of kaempferol.

### Effect of kaempferol on sperm profiles of envenomed rats

Comparing untreated envenomed group to the control, NnV caused substantial (p < 0.05) decrease in the proportion of motile spermatozoa and an increase in slow-progressing sperm cells. In contrast, antivenom and kaempferol treatment enhanced motile spermatozoa significantly (p < 0.05) together with an increase in fast progressive sperm cells. This effect was more pronounced in the envenomed group that received 8 mg/kg of kaempferol treatment (Table 3). Additionally, Table 3 indicates that kaempferol improved the sperm characteristics in a dose-dependent manner, while the venom significantly (p < 0.05) lowered sperm volume and count as observed in the untreated group.

**Table 3:** Sperm parameters of envenomed rats.

Sperm motility (%)					Sperm volume (mL)	Sperm count (10^6^/mL)
Groups	Immotile	Motile	Fast progressive	Slow progressive		
1	30.00 ± 5.77^c^	70.00 ± 5.77^a^	50.00 ± 5.77^a^	20.00 ± 0.00^ab^	12.00 ± 1.32^a^	16.34 ± 0.24^a^
2	81.66 ± 4.40^a^	18.34 ± 4.40^c^	11.66 ± 3.33^d^	6.68 ± 1.66^d^	3.21 ± 0.75^d^	4.31 ± 0.41^d^
3	45.00 ± 2.88^b^	55.00 ± 2.88^b^	40.00 ± 0.00^c^	15.00 ± 2.01^c^	4.31 ± 0.15^c^	5.71 ± 0.21^c^
4	35.00 ± 2.88^bc^	65.00 ± 2.88^ab^	42.33 ± 1.66^b^	22.67 ± 1.67^b^	5.31 ± 0.45^b^	6.41 ± 0.31^b^
5	31.67 ± 4.41^c^	68.33 ± 4.41^b^	43.33 ± 1.66^b^	25.00 ± 2.88^a^	5.51 ± 0.75^b^	6.81 ± 0.41^b^

Data are represented as mean ± SE (n = 5). Mean with different lower-case letter represent significant difference among the groups at p < 0.05 using DMRT.

Group 1: Injected with saline (Control), Group 2: Envenomed not treated (Venom control), Group 3: Envenomed and treated with 0.2 ml of antivenom, Group 4: Envenomed and treated with 4 mg/kg^−1^ of kaempferol, Group 5: Envenomed and treated with 8 mg/kg^−1^ of kaempferol.

### Sperm abnormalities after treatment of envenomed rats with kaempferol

The percentage of sperm abnormalities increased significantly (P < 0.05) in the envenomed untreated rats as compared to the control and envenomed treated rats. However, the detected sperm anomalies were greatly reduced in a dose-dependent manner after treatment with varying dosages of kaempferol. In the envenomed untreated rats, sperm abnormalities such as amorphous heads, banana shapes, folded sperm, short hooks, and no hooks were common while unique sperm abnormalities that were identified were double heads and long, sickled hooks (Table 4).

**Table 4:** Sperm abnormalities after treatment of envenomed rats with kaempferol.

Sperm abnormalities	Group 1	Group 2	Group 3	Group 4	Group 5
Amorphous head	3.66 ± 0.66^d^	68.00 ± 2.00^a^	42.12 ± 0.25^b^	38.33 ± 0.11^b^	30.16 ± 0.36^c^
Banana shape	6.40 ± 1.35^d^	52.21 ± 1.23^a^	33.11 ± 0.62^b^	30.26 ± 0.22^b^	25.10 ± 0.22^c^
Double tails	1.20 ± 0.00^d^	35.23 ± 1.40^a^	25.20 ± 0.34^b^	24.12 ± 0.14^b^	18.00 ± 1.00^c^
Folded sperm	2.11 ± 0.20^e^	54.21 ± 0.30^a^	34.11 ± 0.20^b^	28.80 ± 0.50^c^	22.41 ± 0.31^d^
Long and sickled hook	0.00 ± 0.00^e^	32.42 ± 0.21^a^	22.12 ± 0.21^b^	18.32 ± 0.30^c^	12.31 ± 0.20^d^
Double head	0.00 ± 0.00^e^	34.23 ± 0.23^a^	28.33 ± 0.10^b^	24.21 ± 0.23^c^	22.10 ± 0.10^d^
Short hook	5.41 ± 0.12^d^	54.42 ± 0.41^a^	34.21 ± 0.21^b^	27.15 ± 0.57^bc^	24.51 ± 0.22^c^
Wrong tail attachment	4.23 ± 0.31^e^	40.22 ± 0.32^a^	28.08 ± 0.11^b^	25.10 ± 1.02^c^	20.12 ± 0.22^d^
Pin head	31.22 ± 0.21^d^	29.03 ± 0.31^a^	20.01 ± 0.11^b^	18.22 ± 0.12^c^	14.04 ± 0.12^e^
No hook	6.21 ± 0.36^e^	58.42 ± 1.00^a^	48.25 ± 0.21^b^	39.52 ± 1.10^c^	30.23 ± 0.24^d^
Wrong-angled hook	5.24 ± 0.55^e^	41.34 ± 1.22^a^	25.14 ± 1.11^b^	22.40 ± 1.33^c^	16.14 ± 0.12^d^
Total abnormal cells	65.68 ± 2.88^e^	499.73 ± 5.46^a^	340.68 ± 3.28^b^	296.43 ± 2.34^c^	235.12 ± 4.12^d^
Percentage abnormalities	6.57 ± 1.27^e^	49.97 ± 3.20^a^	34.06 ± 1.66^b^	29.64 ± 3.40^c^	23.51 ± 3.32^d^

Data are represented as mean ± SE (n =5 ). Mean with different lower-case letter represent significant difference among the groups at p < 0.05 using DMRT. Mean ± S.E are fractions of the 1000 sperm cells assessed.

Group 1: Injected with saline (Control), Group 2: Envenomed not treated (Venom control), Group 3: Envenomed and treated with 0.2 ml of antivenom, Group 4: Envenomed and treated with 4 mg/kg^−1^ of kaempferol, Group 5: Envenomed and treated with 8 mg/kg^−1^ of kaempferol.

### Concentration of male sex hormones in envenomed rats treated with kaempferol

**Figure 1:**
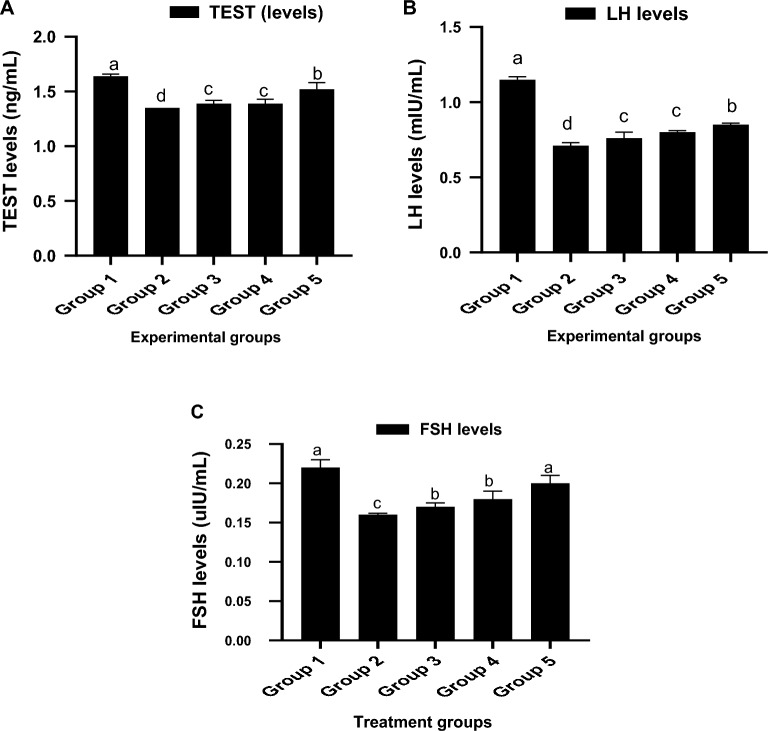
Concentrations of serum male reproductive hormones in envenomed rats treated with kaempferol. Data are represented as mean ± SE (n = 10). Bar with different lower-case letter represent significant difference among the groups at p < 0.05 using DMRT. *FSH* follicle stimulating hormone, *TEST* testosterone hormone, *LH* luteinizing hormone. Group 1: Injected with saline (Control), Group 2: Envenomed not treated (Venom control), Group 3: Envenomed and treated with 0.2 ml of antivenom, Group 4: Envenomed and treated with 4 mg/kg^−1^ of kaempferol, Group 5: Envenomed and treated with 8 mg/kg^−1^ of kaempferol.

In comparison to the control group, the serum concentrations of testosterone (TEST) and luteinizing hormone (LH) were considerably (p < 0.05) lower in the envenomed untreated rats (Fig. 1A,B). Nevertheless, the LH and TEST concentrations in the blood of the envenomed treated rats was normalized after receiving kaempferol treatment. Follicle stimulating hormone (FSH) concentration in the venom control also significantly decreased (p < 0.05) in comparison to the control. The administration of 8 mg/kg of kaempferol resulted in a considerable increase in FSH levels (Fig. 1C).

### Oxidative stress status of the testes and epididymis

#### Malondialdehyde (MDA) levels

**Figure 2:**
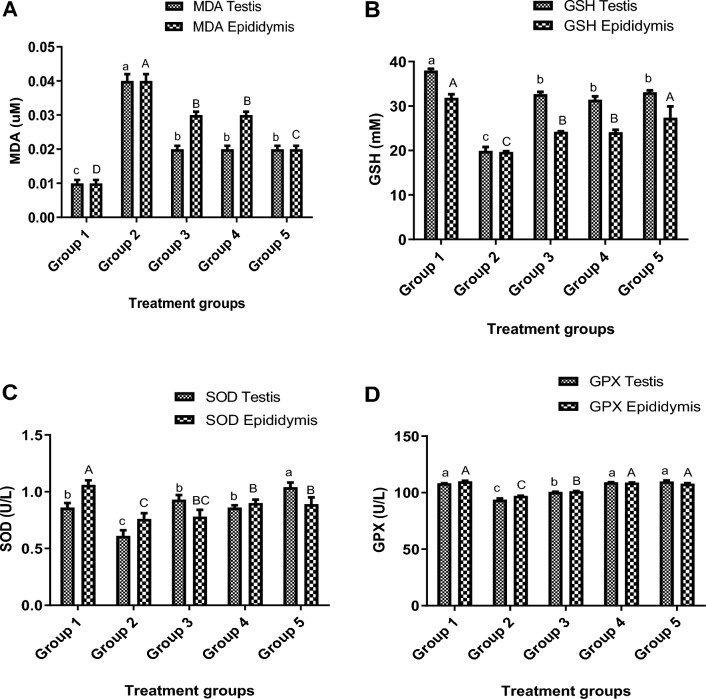
Stress profiles of the testis and epididymis of envenomed rats treated with kaempferol. Data are represented as mean ± SE (n = 5). Bar with different lower-case letter represent significant difference in stress profile of testis among the groups at p < 0.05 using DMRT. Bar with different upper-case letter represent significant difference in stress profile of epididymis among the groups at p < 0.05 using DMRT. *MDA* malondialdehyde, *GSH* glutathione, *SOD* superoxide dismutase, *GPX* glutathione peroxidase. Group 1: Injected with saline (Control), Group 2: Envenomed not treated (Venom control), Group 3: Envenomed and treated with 0.2 ml of antivenom, Group 4: Envenomed and treated with 4 mg/kg^−1^ of kaempferol, Group 5: Envenomed and treated with 8 mg/kg^−1^ of kaempferol.

In comparison to the control and envenomed treated groups, the levels of MDA in the testes and epididymis of the envenomed untreated rats were considerably (p < 0.05) greater. When compared to the envenomed groups treated with 4 mg/kg and antivenom, respectively, the envenomed group treated with 8 mg/kg of kaempferol recorded a significantly (p < 0.05) lower value (Fig. 2A).

#### Reduced glutathione (GSH) levels

The group envenomed with NnV and not treated post-envenomation had considerably (p < 0.05) lower GSH levels when compared to the control group. Nevertheless, the antivenom treatment and different kaempferol doses substantially (p < 0.05) upregulated the GSH levels in the envenomed treated groups. However, the envenomed group treated with 8 mg/kg of kaempferol showed a substantial (p < 0.05) increase compared to the envenomed groups treated with 4 mg/kg of kaempferol and antivenom, respectively (Fig. 2B).

#### Superoxide dismutase (SOD) activity

The testis and epididymis of envenomed untreated rats exhibited considerably higher levels of SOD activity compared to the control group. In contrast to the envenomed untreated group, antivenom therapy and various kaempferol concentrations significantly (p < 0.05) increased the activity of SOD in the reproductive organs. When compared to groups treated with 4 mg/kg of kaempferol and antivenom, the envenomed group treated with 8 mg/kg of kaempferol recorded a significantly (p < 0.05) greater value (Fig. 2C).

#### Glutathione peroxidase (GPX) activity

In the testis and epididymis of untreated rats, the NnV significantly (p < 0.05) reduced the GPX activity in comparison to the control. In contrast to groups treated with antivenom, the GPX activity in the envenomed treated groups was increased by varying dosages of kaempferol. The envenomed groups treated with 4 and 8 mg/kg of kaempferol did not significantly (p < 0.05) differ in the activity of GPX in the testis and epididymis (Fig. 2D).

### Inflammatory status of the testis and epididymis

#### Nitric oxide (NO) levels

**Figure 3:**
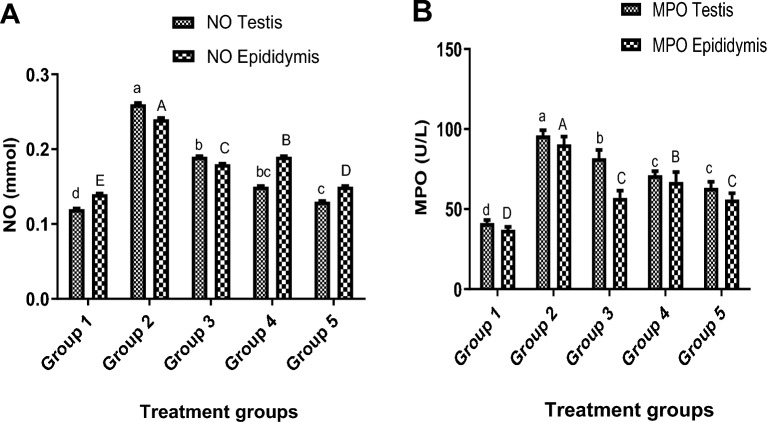
Inflammatory profiles of the testis and epididymis of envenomed rats treated with kaempferol. Data are represented as mean ± SE (n = 5). Bar with different lower-case letter represent significant difference in inflammatory markers of the testis among the groups at p < 0.05 using DMRT. Bar with different upper-case letter represent significant difference in inflammatory markers of epididymis among the groups at p < 0.05. *NO* nitric oxide, *MPO* myeloperoxidase. Group 1: Injected with saline (Control), Group 2: Envenomed not treated (Venom control), Group 3: Envenomed and treated with 0.2 ml of antivenom, Group 4: Envenomed and treated with 4 mg/kg^−1^ of kaempferol, Group 5: Envenomed and treated with 8 mg/kg^−1^ of kaempferol.

The testis and epididymis of the envenomed untreated group showed higher levels of NO compared to the control and envenomed treated rats. However, the NO levels were significantly (p < 0.05) repressed when antivenom and various doses of kaempferol were administered to envenomed rats. The group administered with 8 mg/kg of kaempferol recorded a notable decrease compared to other treatment groups (Fig. 3A).

#### Myeloperoxidase (MPO) activity

In the envenomed untreated group, NnV significantly (p < 0.05) increased the MPO activity in the testis and epididymis compared to other experimental groups. On the other hand, kaempferol administration resulted in a significant (p < 0.05) dose-dependent decrease in MPO activity, with the group administered with 8 mg/kg of kaempferol showing the greatest effect (Fig. 3B).

### Histological evaluation

#### Testes

**Figure 4:**
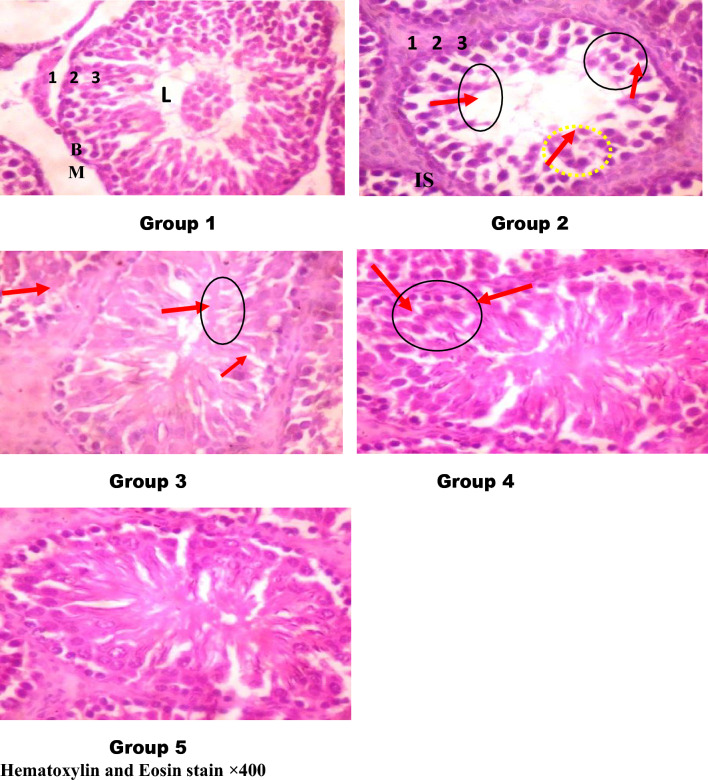
Photomicrographs of the testicular histomorphology of envenomed treated rats. *BM* The seminiferous epithelium and basement membrane, *L* The lumen, *IS* interstitial space containing interstitial cells, *SG* spermatogonium (1^0^,2^0^,3^0^). Group 1 (control): showed normal seminiferous tubules lined with spermatogenic cell layers with their lumen (L) with no observable lesion. Group 2 (venom control): revealed severe testicular degeneration within the spermatogenic cell layers, hyperplasia of Leydig cells within edematous and atypia within some spermatogenic cells (red arrows). Groups 3 (venom/antivenom): showed mild testicular degeneration within the spermatogenic cell layers (red arrow) with a core of desquamative spermatogenic cells. Groups 4 (venom/4 mg/kg kaempferol): showed mild testicular degeneration within the spermatogenic cell layers (arrows) with a core of desquamative spermatogenic cells, Groups 5 (venom/4 mg/kg kaempferol): No observable pathomorphology was seen.

The control rats' plates showed normal seminiferous tubules lined with spermatogenic cell layers with their lumen and no visible tissue lesion was noticed. In contrast, NnV caused severe testicular degeneration within the spermatogenic cell layers along with hyperplasia of Leydig cells combined with edematous and atypia of the spermatogenic cells in the envenomed untreated rats (Fig. 4, plate 2). The envenomed group treated with antivenom exhibited moderate testicular degeneration within the spermatogenic cell layers (Fig. 4, plate 3). On the other hand, a core of desquamative spermatogenic cells was observed in the spermatogenic cell layers of the envenomed group that received 4 mg/kg of kaempferol treatment, exhibiting a slight degeneration of the testicles (Fig. 4, plate 4), whereas the envenomed group treated with 8 mg/kg of kaempferol showed no observable histomorphology (Fig. 4, plate 5).

#### Epididymis

**Figure 5:**
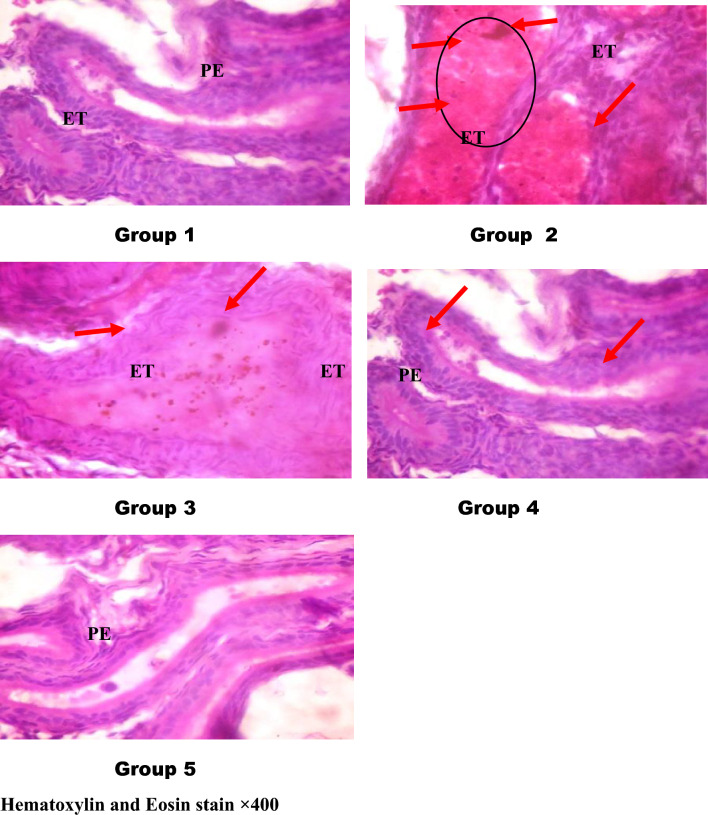
Photomicrographs of the epididymal histomorphology of envenomed treated rats. *ET* epididymal tubules, *PE* pseudostratified columnar epithelium, and arrows: hemorrhage in the lumen. Group 1 (control): showed regular and circular tubules with a pseudostratified columnar epithelium that exhibits stereocilia with no alteration in epididymal structural integrity, Group 2 (venom control): revealed extra tubular space contains blood vessels in the interstitial connective tissue and evidence of hemorrhage (red arrows). Groups 3 (venom/antivenom): The epithelium was separated from the connective tissue with extra tubular space containing blood vessels in the interstitial connective tissue (red arrows), Groups 4 (venom/4 mg/kg kaempferol): There is a vacuole formation within pseudostratified epithelium with no marked histological alteration, Groups 4 (venom/4 mg/kg kaempferol): There is a vacuole formation within pseudostratified epithelium with no observable marked histological alteration.

The control rats' epididymis (Fig. 5, plate 1) displayed normal, circular tubules together with a pseudostratified columnar epithelium that displayed stereocilia, all without compromising the structural integrity of the epididymis. On the other hand, the plates of envenomed untreated rats (Fig. 5, plate 2) showed extra tubular space containing blood vessels in the interstitial connective tissue with evidence of hemorrhage. The antivenom-treated envenomed group (Fig. 5, plate 3) displayed an epithelium that was isolated from the connective tissue and had extra tubular space in the interstitial connective tissue that contained blood vessels. Meanwhile, vacuole development within pseudostratified epithelium was observed in the envenomed groups treated with 4 and 8 mg/kg of kaempferol (Fig. 5, plate 4 and 5), with no marked histological alteration, indicating the compound ameliorated the structural alterations caused by NnV on the organ tissues.

## Discussion

In tropical and underdeveloped nations across the world, cobra envenomation poses a serious public health risk, leading to elevated rates of morbidity and fatality^24^. The cytotoxic components of the venom are responsible for significant detrimental effects on organ systems through cytolytic activity via pore formation on cell membranes, and disruption of membrane lipid bilayer integrity, thus initiating cell death in cobra envenomed victims^25^. However, timely treatment intervention using specific antivenom is crucial for neutralizing the venom toxins and to prevent further damage to the organs of snakebite victims.

Plant-derived products have been a recent target of research for their numerous health benefits as a potential basis for the development of new drugs because of their minimal clinical side effects and easy accessibility. This study aims to evaluates the reproductive and physiopathological disorders caused by NnV and assess the potential ameliorating ability of kaempferol against reproductive toxicity post snakebite envenoming. In this study, NnV caused high mortality in group envenomed and not treated post-envenomation. However, kaempferol at 8 mg/kg dose protected the envenomed animals and no death was recorded indicating the compound neutralized the venom toxins and halted further systemic fatalities that could result to death. The observation at this dose of the compound was consistent with our previous study using a viper venom^21^. NnV induced significant decrease in testicular weight and testiculosomatic index of the envenomed untreated rats, an evidence of testis toxicity attributed to the detrimental effect of the venom toxins by initiating necrotic activity resulting in testicular weight loss as earlier reported^11,26^. However, there was significant improvement in the organs weight and organo-somatic index of the envenomed groups treated with 4 and 8 mg/kg of kaempferol.

Envenoming by snake allows the diffusion of the venom toxins into the bloodstream and transported to different organs to exert its destructive effects. Spermatozoa are produced in the testis and stored in the epididymis which are vital reproductive organs. These sperm cells are sensitive and susceptible to venom toxic components^27^. As a result, NnV toxins altered spermatogenesis in the testes and decimated the sperm cells stored in the epididymis as significant decline was noticed in motile sperm, fast progressive cell, volume and count in the envenomed untreated rats attributed to cytotoxic constituents such as phospholipase A2 (PLA2) present in NnV. PLA2 are cytotoxins that causes the hydrolyzation of sn-2 fatty acid acyl ester bond of phosphoglycerides to free fatty acid and lysophospholipids and initiates the disintegration of plasma membranes phospholipids which affected the reproductive organs and contributed immensely to the depreciated sperm parameters^28,29^.

Studies have documented these toxic effects attributed to cytotoxic PLA2 on sperm motility and sperm acrosome reactions^30^. Also, our previous studies have reported such damaging effects of NnV^11^ and *Echis ocellatus* venom^16^ on sperm parameters in male envenomed rats. Results obtained in the untreated envenomed group showed that NnV could cause infertility in snake envenomed victims if proper treatment is not administered in time as previously posited^11^. Kaempferol displayed exceptional neutralizing ability to combat the cytotoxic activity of NnV toxins in the envenomed treated groups and significantly elevated the sperm parameters to an appreciable level in a dose dependent manner compared to the reference drug (antivenom). Motile viable sperm cells are key for fertilization and the significant improvement in sperm motility and fast progressive sperm cells post kaempferol treatment is suggestive that the compound could serve as fertility booster and increases the chances of fertilization.

Defective sperm cells or altered acrosome reaction that emanates from spermatozoa abnormalities are one of the major causes of infertility. NnV caused intense harmful effect on the spermatozoa inducing various sperm abnormalities prominent in the envenomed untreated rats which aligned with previous studies^11,31^. This could be as a result of upsurge in free radical production caused by NnV toxins especially the hydroxyl (OH−) interacting with the DNA’s sperm head, proteins and lipids to induce oxidative damage^11^. However, treatment with kaempferol decreased the observed sperm anomalies in the envenomed treated groups when compared to the envenomed untreated rats. This ameliorative effect of kaempferol can be attributed to its antioxidant and cytoprotective properties.

Sex hormones such as testosterone, FSH and LH are vital hormones for the initiation and completion of spermatogenesis and the development of reproductive tissues in males^32^. NnV caused hormonal disorder and decreases the sera concentrations of these important sex hormones in the envenomed untreated groups which corroborated our previous study^11^. The venom toxins may have caused a detrimental impact on the biological process of sex hormones production, thereby interfering with the hypothalamic-pituitary–gonadal axis that controls sperm production in the testis^33^. Also, the venom toxins could have altered the biosynthesis and secretion processes of male sex hormones by inflicting damaging effects on the Leydig interstitial cells of the testes^34^. This could be responsible for the marked depreciation in sperm counts and volume of envenomed untreated group as observed in this study. The elevated male sex hormones levels after treatment with kaempferol could be that the compound alleviates the damages to the Leydig interstitial cell and boosts hormones synthesis.

Envenomation is often accompanied by the activation of oxidative stress and inflammatory processes and its cascade of events plays a vital role in the clinical pathogenesis after snakebite envenoming^35^. Oxidative stress and inflammation are closely related to the development of local and systemic deleterious effects due to snake envenomation. This study demonstrated that NnV initiated oxidative stress and activates inflammatory response with high expression of diverse endogenous oxidative and inflammatory biomarkers. The venom elevated levels of MDA, NO and MPO activity in the testes and epididymis of envenomed untreated group indicating induced oxidative stress and inflammation. Cells of vital reproductive organs are vulnerable to oxidative stress and inflammation and thus, need sufficient potent antioxidants to eliminate free radical damage produced by the lipid peroxidation chain reaction^36^. Treatment with kaempferol suppressed these biomarkers thus, inhibiting the lipid peroxidation chain reaction and inflammation in the reproductive organs examined. NnV induced oxidative stress as revealed by significant decrease in GSH, GPX and SOD in the testis and epididymis of untreated envenomed rats which supported our previous findings^11^. Suppression of activities of these endogenous antioxidant enzymes is evidence of upsurge in reactive oxygen species (ROS) resulting in oxidant damage^36^. However, treatment with kaempferol enhanced the activity levels of these endogenous antioxidant enzymes which was in tandem with our previous study in viper envenomed rats^21^.

The biochemical results corroborated the histopathological findings as NnV caused severe testicular degeneration within the spermatogenic cell layers while the epididymal tissues revealed extra tubular space contains blood vessels in the interstitial connective tissue and hemorrhage in envenomed untreated rats. These structural defects in the reproductive organs of the group could also be responsible for the depreciation in the sperm parameters as observed in this study. However, kaempferol ameliorated the structural lesions observed in the testis and epididymis of envenomed treated rats compared to the antivenom. Phytochemicals such as flavonoids, terpenoids and alkaloids possesses therapeutic benefits with the ability to strengthen the antioxidant defense system in various organs in the body, including the testis and epididymis thereby influencing their effective functioning, preventing oxidative stress and inflammation^22^. The exact mechanisms of kaempferol in mitigating these systemic disorders may not be ascertained but studies have revealed that multidimensional underlying mechanisms could be responsible for the effectiveness of plants and their bioactive compounds, which include but not limited to anti-inflammatory, antioxidant, firm hydrogen bonding with amides of protein chains, anti-apoptotic, meta-chelating activity, direct elimination of ROS, upregulating the expression of encoding ROS-removing genes and specific neurotrophic mechanisms^22,37,38^.

## Conclusion

The results of this investigation support the antivenom potency of kaempferol against toxicities induced by snake venom on organ systems and point to the compound's potential clinical utility in the treatment and management of reproductive pathophysiological conditions that may arise from snakebite envenomation. A comprehensive approach is necessary for the effective management of snake envenomation, including early detection, prevention, and timely availability to a suitable and efficient antivenom treatment. If further investigated, kaempferol may offer fresh chances for successful therapeutic discovery. Also, it might be utilized in combination with serum antivenom as effective intervention to lessen the organs system disorders that could manifest post snakebite envenoming.

## Materials and methods

### Venom and antivenom

Lyophilized NnV was obtained from the University of Ibadan's Department of Zoology's serpentarium in Nigeria and kept in the lab at 4 °C. The research employs EchiTAb-Plus ICP polyclonal antivenom as the reference drug.

### Kaempferol

Kaempferol (MF: C15H10O6, MW: 286.24 g/mol) in an exact dosage of 200 mg was obtained from Sigma-Aldrich®, USA, St Louis, Missouri, USA, and kept at 20 °C until needed.

### Ethics statement

The animal procedures followed the regulations of the University of Ibadan-Animal Care and Use Research Ethics Committee (UI-ACUREC). All animal experimental protocols were approved by University of Ibadan-animal care and use research ethics committee with approval number UI-ACUREC/19/0030. Furthermore, the experiment was carried out in compliance with the revised ARRIVE guidelines 2.0.

### Animals

Fifty male albino Wistar rats, weighing between 100 and 140 g were procured from Osun State University's Central Animal Facility in Osogbo, Nigeria. The animals were transferred in transparent plastic cages with good ventilation to the Department of Zoology Laboratory at Osun State University. There, they were allowed to acclimate for two weeks to standard ambient conditions, which included 25 ± 2 °C and 50 ± 15% relative humidity. The rats had unrestricted access to clean water and conventional rat food during a 12-h cycle of light and dark.

#### Study design and procedures

Five groups of ten rats apiece were created via random assignment of the animals. At exactly 8:00 am, saline was injected into group 1 as a control while groups 2 through 5 received a single intraperitoneal injection of 1.0 mg/kg^−1^ (LD^50^) of NnV^10^. Group 2 received no treatment, while treatment commenced 30 min post envenomation in groups 3, 4, and 5 which received intraperitoneal injections of polyvalent antivenom (0.2 ml) and 4 and 8 mg/kg of kaempferol, respectively in accordance with previous studies^10,19,21^. The envenomed animals in groups 3, 4 and 5 were treated for seven days in a row for effective neutralization of the venom toxins. In accordance with the completion of rat spermatogenesis, the experimental period lasted 50 days^39^ after envenomation. Throughout the investigation, animals were observed for clinical indications of toxicity and mortality.

#### Body weight changes

The body weight changes of the experimental animals were determined by measuring the weight of each animal pre-venom injection on day 1 as initial weight and at termination before they were sacrificed as terminal weight. Body weight changes was calculated using the formula:

$$Body\,weight\,gain=\frac{Terminal\,weight\,of\,rats-Initial\,weight\,of\,rats}{Initial\,weight\,of\,rats} \times 100$$

#### Blood and organ sample collection

Blood samples were collected from each experimental rats using heparinised capillary tubes into plain bottles through retro-orbital sinus punctuation. The blood was centrifuged at 380×*g* for 10 min to obtain serum for hormonal assays. The rats were thereafter sacrificed based on guides^40^. The testes and cauda epididymis were surgically removed and weighed. The right epididymis was used for analysis of sperm parameters while the left epididymis and testes were divided into two portions; a portion was used for biochemical assays, while other portions were fixed in 10% formalin for histopathological investigations. The relative testes weight was determined using the formula:

$$Relative\,organ\,weight=\frac{Organ\,weight }{Termimal\,body\,weight }\times 100$$

### Assessment of epididymal sperm parameters

#### Sperm volume

The sperm volume was determined by immersing the epididymis in 5 ml normal saline in a measuring cylinder and the volume displaced was taken as the volume of the epididymis^41^.

#### Sperm motility

The right cauda epididymis was placed individually in a petri dish and minced in normal saline (1 ml) to form the sperm suspension. 10 µl of the suspension was dropped on a microscopic slide and observed for motility under the light microscope at a magnification of × 400. Sperm motility was assessed by classifying 200 spermatozoa into two categories, motile and immotile spermatozoa. Three sperm classes were categorized as motile spermatozoa: rapid progressive, slow progressive and non-progressive spermatozoa^42^.

#### Sperm counts

For sperm count, a further 1:10 serial dilution of the sperm suspension was prepared out of which 10 µl of the dilution was counted under a light microscope at a 400 × magnification with the aid of an improved Neubauer hemocytometer as described by WHO^42^. Sperm count values were multiplied by the dilution factor and recorded as millions per milliliter (10^6^/ml).

#### Sperm morphology

Exactly 450 µl of the sperm suspension was mixed with 50 µl of 1% aqueous eosin Y for 45 min. The stained sperm suspension was used to make a thin smear on a pre-cleaned grease-free microscopic slide. Prepared slides were allowed to air dry and abnormalities were observed in 250 spermatozoa with four replicates in each rat at a magnification of × 1000^43^.

### Measurement of male reproductive hormones

The sera obtained from the experimental rats were analyzed to determine the concentrations of testosterone, Follicle Stimulating Hormone (FSH), and Luteinizing Hormone (LH) using the Enzyme-Linked Immunosorbent Assay (ELISA) kits (Biocheck, South San Francisco, CA, USA).

### Biochemical profiles of the testis and epididymis tissues

#### Preparation of homogenates

Using a Teflon homogenizer, sections of the testis and epididymis were cut into small pieces and homogenized in four liters of the homogenizing buffer (0.1 M Tris-KCl, pH 7.4). To extract the post mitochondrial fraction, the resultant homogenate was centrifuged at 12,500×*g* for 15 min at 4 °C in a cool centrifuge. After being collected, the supernatant was used for biochemical investigations.

#### Oxidative stress profiles

Lipid peroxidation end product; malondialdehyde (MDA), was measured as thiobarbituric acid reactive substance (TBARS) as described^44^. The levels of reduced glutathione (GSH) were assayed^45^. Superoxide Dismutase (SOD) and glutathione peroxidase (GPX) activities were measured as described by Marklund and Marklund^46^ and Rotruck et al.^47^, respectively.

### Inflammation profiles

The levels of nitric oxide (NO) and myeloperoxidase (MPO) were assessed by procedures described by Green et al*.*^48^ and Granell et al.^49^, respectively.

### Histopathological examination

Sections of the testis and epididymis were processed and examined for structural defects using conventional techniques of paraffin-wax sectioning and hematoxylin–eosin staining^50^.

### Data analysis

The results obtained were expressed as mean ± Standard Error of Mean (SEM). Significant differences between the experimental groups were tested using one-way Analysis of Variance (ANOVA) and Duncan multiple range test of the control and treatment groups. The differences between mean values were considered significant at p < 0.05. Statistical Package for Social Sciences (SPSS, version 25) software produced by IBM Corp. Ltd. was used for data analysis.

## Data Availability

The data sets used and/or analyzed during the current study are available from the corresponding author on reasonable request.
